# Improving quality of life in cancer patients through higher participation and health literacy: study protocol for evaluating the oncological social care project (OSCAR)

**DOI:** 10.1186/s12913-019-4585-0

**Published:** 2019-10-26

**Authors:** Johann Frick, Daniel Schindel, Pimrapat Gebert, Ulrike Grittner, Liane Schenk

**Affiliations:** 1 Institute of Medical Sociology and Rehabilitation Science, Charité – Universitätsmedizin Berlin, Campus Charité Mitte, Charitéplatz 1, 10117 Berlin, Germany; 2 Institute of Biometry and Clinical Epidemiology, Charité – Universitätsmedizin Berlin, Campus Charité Mitte, Charitéplatz 1, 10117 Berlin, Germany; 3 Berlin Institute of Health (BIH), Anna-Louisa-Karsch-Str. 2, 10178 Berlin, Germany

**Keywords:** Social support, Cancer, Quality of life, Patient preferences, Health literacy

## Abstract

**Background:**

Cancer patients experience psychological and social distress due to their medical treatment and social issues. However, continuous and specialized social support is still lacking. In Germany, a group of company health insurance funds has developed an approach to support cancer patients with monthly structured interviews conducted by specially trained Social Care Nurses. The nurses will identify patient needs in order to provide help with medical, personal, and social matters. One aim of the scientific evaluation is to analyze the effect of the consultations on various patient-reported outcomes, especially quality of life. The evaluation concept will be described in this study protocol.

**Methods/design:**

The evaluation is a non-randomized, controlled, multi-center intervention study with a mixed-method design. It consists of three research modules which include primary data from questionnaires, and claims data from the health insurance funds. In Module 1, cancer patients will be recruited to form an intervention group (OSCAR, *n* = 150) and a control group (*n* = 200) in four study centers for a period of 1 year. One baseline and three follow-up questionnaires will be conducted to survey the patient-reported outcomes. Relevant secondary outcomes are health literacy, participation, and physician-patient communication. In Module 2, claims data will be used to analyze cost effects and thereby assess effectivity and hospitalization. Module 3 will involve a qualitative analysis of project diaries kept by the Social Care Nurses. The diaries will record the nurses’ practical experiences and the benefits of deploying OSCAR across the German healthcare system.

**Discussion:**

OSCAR is an innovative way of providing cancer patients with continuous support to improve their quality of life. The evaluation concept aims to assess the effects of the monthly consultations by the Social Care Nurses on the patients, and will use a mixed-method design. The results are important for assessing the transferability of OSCAR to the healthcare system as a whole.

**Trial registration:**

German Clinical Trials Register (DRKS-ID: DRKS00013640). Registered 29 December 2017.

## Background

### Demand for social support for cancer patients

Cancer cause a high number of incident cases in Germany every year (229,920 women and 252,550 men in 2013) [1]. Due to the progression of the diseases, patients and their relatives experience distress caused by medical treatment and social issues [2, 3]. The misunderstanding of the disease can lead to inappropriate self-assessments of one’s prognosis and a choice of unsuitable medical treatments [4]. Simultaneously, patients receive overtreatment even though they have an incurable disease [5]. In these situations, palliative care could be appropriate for improving quality of live [6]. Enhancing palliative care can help patients to find suitable medical treatments that can increase quality of life. However, the specific needs of cancer patients are not taken into adequate account by the healthcare system because of a lack of palliative care [7]. Currently, patient navigation programs are used to help patients with medical, nursing, psychological, and healthcare-related issues. Recent studies have attempted to evaluate the impact of these programs (such as *Onkolotse* by the Saxon Cancer Society) [8, 9]. In this context, patient-reported outcomes (PROs) are very important for examining the subjective benefit of interventions from the patient’s perspective, with the focus on relevant outcomes (e.g. quality of life) [10].

### Development of the OSCAR project

The Oncological Social Care Project (OSCAR) was developed by the German company health insurance fund Pronova BKK. The concept is based on the Saxon Cancer Society’s *Onkolotse* navigation program for cancer patients. An essential component of OSCAR is the additional social support provided by the Social Care Nurses (SCNs), who aim to identify deficits in healthcare utilization by cancer patients and support them in relevant areas (e.g. the medical treatment of the cancer, psychosocial support for anxiety, practical tips regarding social security services, and organization of rehabilitation). Moreover, the SCNs are regular and accessible contact partners in a complex and fragmented multidisciplinary treatment process. Each patient is accompanied for 1 year, regardless of whether they are being treated as inpatients or outpatients, or are currently not receiving any therapy. The intervention aims to access and improve the quality of life for patients with advanced cancer and a poor prognosis. It uses twelve structured interviews over the course of 1 year. The SCNs received training from the Saxon Cancer Society. OSCAR is implemented by five SCNs at four locations.

## Methods

**Fig. 1:**
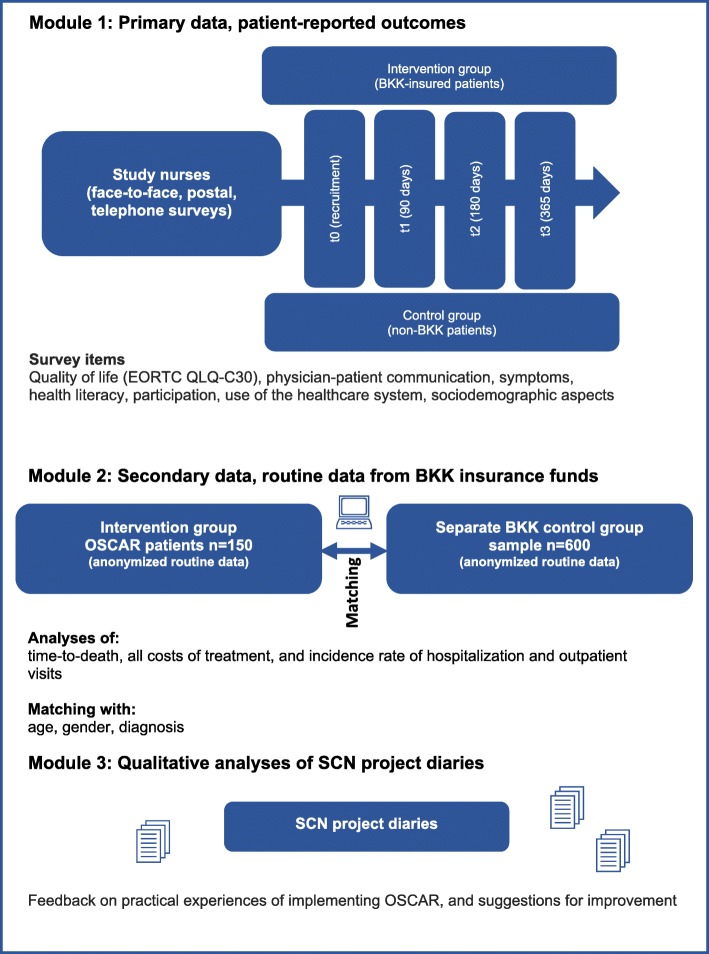
Modules in the OSCAR evaluation concept

The OSCAR evaluation is a mixed-method study consisting of three research modules. Modules 1 and 2 involve a non-randomized, controlled, multi-center intervention study which compares primary data from regularly conducted questionnaires and claims data from German statutory health insurance funds in the intervention and control groups. In addition, a qualitative content analysis is planned in module 3 and will be based on the project diaries completed by each SCN. The analysis will assess OSCAR’s effectivity and the benefit of implementing it in the German healthcare system (Fig. 1).

### Study objectives

Primary objective:

To compare quality of life over study period between the intervention group (patients who had met an SCN) and the control group (Module 1).

Secondary objective:

1. To compare the health literacy, participation, and physician-patient communication in the intervention and control groups (Module 1)
2. To explore the associations between quality of life and health literacy, participation, and physician-patient communication (Module 1)
3. To compare the incidence rate of outpatient visits in both groups (Modules 1 and 2)
4. To compare the healthcare costs in both groups (Module 2)
5. To assess the SCN’s practical experiences (Module 3)

### Inclusion and exclusion criteria for participation in the evaluation survey in modules 1 and 2

Inclusion criteria:

- Aged ≥18 years
- At least a combination of one type of cancer and one operation and procedure, as defined by the ICD and OPS codes listed in Additional file 1

Exclusion criteria:

- Advanced dementia
- Acute addictions

### Module 1: primary data, patient-reported outcomes

#### Participant recruitment

The recruitment was carried out in four different hospitals with oncology departments across three cities in two states in Germany. Two of the hospitals are located in Berlin, while the other two are in Leverkusen and Duisburg.

In all four study sites, the SCNs recruited BKK-insured patients for the intervention group by obtaining informed consent. The SCNs are responsible for the monthly consultations to support the patients in the intervention group. Patients who are insured with a different provider were recruited by Study Nurses (SNs) for the control group. The SNs’ main task is to conduct the evaluation questionnaires in both study groups up to four times within 1 year. The evaluation’s baseline survey will be conducted after recruitment (t0) and followed up after 90 (t1), 180 (t2), and 365 (t3) days. The patients can choose between personal, telephone, and postal interviews, depending on their health status and which method is most comfortable for them. Dividing responsibilities between SCNs and SNs is necessary to ensure the independence of the evaluation surveys. Patients in both groups were recruited at the same study sites between February 2018 and the end of February 2019.

#### Questionnaire

The items for the evaluation questionnaire are listed in Table 1:

**Table 1:** Items in the evaluation questionnaire

Item	Description
Quality of Life of Cancer Patients (EORTC QLQ-C30) [11]	The EORTC QLQ-C30 is a questionnaire for measuring health-related quality of life, and is provided by the European Organisation for Research and Treatment of Cancer. Patients are asked to answer 30 questions, which address e.g. physical, emotional, cognitive, and social issues. 28 items have four response options. Two items for the global health status and the quality of life have a seven-point scale. The EORTC QLQ-C30 consists of five functional scales and three symptom scales. In addition, a global health status (QoL) scale as well as six single items. All of the scales and the single items will use a linear transformation to standardize the raw score. The scores range from 0 to 100.
Illness Perception Questionnaire (IPQ) [12]	The IPQ consists of 64 items on 8 scales, and measures the individual’s beliefs and feeling about their illness. The illness coherence subscale consists of five items and is used for the evaluation questionnaire in this study. The scores will be summed and divided by the number of items.
Patient Reaction Assessment (PRA-D) [13]	The PRA-D is a self-description instrument to record the patient-perceived quality of the relationship between patients and physicians. In this study, we adapted five communication questions to a five-point Likert scale (from 1 = “I strongly disagree” to 5 = “I strongly agree”).
German modified version of the Autonomy Preference Index (API-Dm) [14]	The API-Dm measures patients’ preferences for participation and information regarding medical decisions. Patients are asked to answer eleven questions using a four-point Likert scale (from 0 = “strongly disagree” to 4 = “strongly agree”). The information preference consists of seven items, with scores ranging from 0 to 28. The participation preference consists of four items, with scores ranging from 0 to 16. The total score of each patients’ preference will be transformed into scores ranging from 0 to 100.
Decisional Conflict Scale (DCS-10) [15]	The DCS-10 is a self-reported questionnaire, which allows an evaluation of decision conflicts among patients. The short version of the DSC comprises ten items grouped into four subscales: uncertainty, information, values clarity, and support decision. Each item is measured on a three-point Likert scale (0 = “yes”, 2 = “unsure”, 4 = “no”). Scores range from 0 (no decision conflict) to 100 (extremely high decision conflict).
European Health Literacy Survey (HLS-EU-Q6) [16]	The HLS-EU-Q6 is the short version of the 47-question European Health Literacy Survey. It is divided into three areas: healthcare, disease prevention, and health promotion. Each of the six questions has five possible responses. A mean score is calculated and allocated to one of three categories: insufficient health literacy (range 1–2), problematic health literacy (2–3), and sufficient health literacy (3–4).
Medical Care Utilization [17]	Medical care will be assessed by the frequency of utilization of different therapies and physician groups.
Oslo Social Support Scale (OSSS-3) [18]	The OSSS-3 is a brief measurement of social support. It consists of three questions that address the number of close people, interest and concern from other people, and practical help from neighbors. The scores range from 3 to 14 and will be categorized into three groups: 3–8 indicates poor support, 9–11 shows moderate support, and 12–14 reflects strong support.

Further instruments are used to measure sociodemographic variables, especially with regard to age, sex, migration status, and social status [19–21]. Finally, participants in the intervention group will be asked to evaluate their contact with the SCN for the last 3 months. The items are related to specific consultation topics and whether or not the support was useful for the respective topic. Participants will also be asked about the quality and quantity of contact with the SCN.

### Statistics

#### Sample size calculation

A sample size of 100 participants in the intervention group and 150 in the control group will reach a power of 80% at a two-sided level of significance of 5% to detect a difference in the EORTC QLQ-C30 (scores range from 0 to 100) with a Cohen’s d effect size of 0.4 (mean difference of ten scores and standard deviation (SD) of 25 scores) [11]. Given that the effect size might be smaller due to a lower mean difference or higher SD, and that the severity of cancer can cause higher dropout rates, we aim to include 150 participants in the intervention group and 200 in the control group (so a total sample size of 350 participants).

The sample size calculation based on the t-test was used in spite of the intended analysis with a baseline-adjusted repeated-measures linear mixed model (ANCOVA, three-level random intercept model to account for repeated measures in patients and clustering in centers). It can be shown [22] that a conservative approach for estimating sample sizes has the same power as a t-test with n subjects where p is the variance deflation factor, calculated by the correlation of baseline and follow-up measures. Assuming the worst case of *p* = 0 leads to the sample size based on the t-test.

#### Statistical analysis plan

All statistical tests are performed using Stata IC15 (StataCorp, 2017, College Station, TX, USA). The primary hypothesis will be tested at a two-sided significance level of α = 0.05. All secondary hypotheses will be tested within an exploratory framework.

Descriptive statistics and the number of participants reflected in the calculation (n) will be presented in each group. For continuous variables, mean with SD for normal distribution and median with interquartile range (IQR) for other distribution variables will be presented. For categorical data, frequencies and percentages will be displayed for each category. Graphic methods such as box plots and line graphs will be used for visualizing the data.

The comparison of EORTC QLQ-C30 (global health status as a primary outcome), physician-patient communication (scores), health competence (scores), participation (scores), and knowledge (scores) over the study period between the groups will be reported as mean and 95% CI, and performed using a linear mixed model (LMM) with three levels over all available time points. Random intercepts for the patient ID and for the study clinics are included in the model to account for the cluster structure of the data. To keep selection bias to a minimum, we will develop a propensity score for adjusting baseline factors. The propensity score will be used with the inverse probability of treatment weighting (IPTW) method because the results from this method are similar to a randomized trial [23, 24].

The number of outpatient visits will be counted and the study time will be calculated over the follow-up time for each patient. The incidence rate of outpatient visits per person-time will be presented, and Poisson regression will be used to compare the incidence rate between groups.

All outcomes will be analyzed using modified intent-to-treat populations including all subjects who receive at least one consultation from an SCN and for whom at least the first follow-up assessment at Visit 1 (at 3 months) is available.

#### Dropouts and missing data

Reasons for dropouts will be documented and reported. If patients are alive and missings are assumed to be missing at random (MAR), we will use multiple imputation methods based on ten imputed data sets [25] with multiple imputation by chained equations (MICE).

### Module 2: secondary data, claims data from health insurance funds

#### Design and participants

Another part of the OSCAR evaluation involves analyzing claims data from the BKK health insurance funds. In contrast to the patient-reported parameters in Module 1, the claims data will make it possible to analyze objective parameters. This kind of data is a valid and relevant source, and has been used in many healthcare research projects in Germany. The data from Modules 1 and 2 will not be linked. If available, the secondary data will be analyzed for the period up to 12 months before and after enrollment.

#### Inclusion and exclusion criteria

This data is only available for the patients in the intervention group, who have health insurance with BKK (*n* = 150). A new, independent and anonymous control group will therefore be drawn from BKK-insured patients who did not take part in the OSCAR intervention (*n* = 600). All included patients must meet the inclusion and exclusion criteria mentioned above. Furthermore, the patients in the control group should be treated in hospitals comparable to those where the patients in the intervention group were treated. In addition, the control group will be matched with the intervention group by age, gender, and diagnosis.

#### Variables

The following aspects are of interest: time-to-death, all costs of treatment, and the incidence rate of hospitalization and outpatient visits.

#### Statistical analysis plan

Paired t-test or Wilcoxon signed rank test will be performed to compare the costs of treatment per month. The generalized estimating equation (GEE) for Poisson regression will be applied to compare the incidence rate of hospitalization and outpatient visits between intervention and matched controls. Kaplan-Meier curve and Cox regression analysis will be performed to compare time-to-death between the groups.

### Module 3: qualitative analyses of SCN project diaries

#### Design and participants

The qualitative analyses of project diaries provide important insights into the work of the SCNs. The nurses will be asked to record their feedback concerning positive aspects of the OSCAR program, as well as opportunities for further improvements with regard to the transferability of OSCAR to the regular healthcare system. For each patient, the SCNs will record the number of additional consultations between two planned monthly consultations, and any recommendations to visit partners in their supply network.

#### Analysis plan

The following aspects are of interest for the qualitative content analysis: acceptance of OSCAR among the patients and among the participating hospitals; number of additional consultations for patients; subjective feedback and reasons for early program termination by the patient; barriers to consultations.

## Discussion

This study aims to evaluate whether the OSCAR program can improve quality of life for patients with advanced cancers and a poor prognosis by enhancing health literacy and participation in therapy planning through continued supportive care from an SCN. A variety of approaches currently exist for developing and evaluating navigation programs for cancer patients. One example is the *Onkolotse* project from the Saxon Cancer Society. It involves certified nurses, psychologists, and social workers, who having contact with cancer patients about 20 times within 1 year. The evaluation of this project focuses on the number of hospital admissions and psychological stress [8]. Nurses from the non-profit association Group Health contact patients weekly via telephone, and meet with them face-to-face at least once [26]. The nurses aim to develop strategies that address distress and focus on quality of life. In another project, experienced nurses attempt to identify patient needs with three different assessment tools and personal conversations [27]. The endpoints in this study are e.g. patient satisfaction, acceptance of the nurses’ advice, and the use of support services for cancer.

Nevertheless, there is a lack of implementation of these approaches in Germany. OSCAR was developed to provide patients with a year of regular support in matters regarding their cancer. This is a useful addition to existing structures such as the social services provided by hospitals, which address the immediate needs of patients after hospital discharge. Improving the continuity of care and building patient competences are important for cancer patients in a fragmented healthcare system. Moreover, suitable healthcare structures and contract frameworks are necessary for delivering low-threshold access to palliative care and supporting consultations.

Experiences gathered with OSCAR could be transferred to other chronic somatic diseases which are associated with severe physical and psychological distress in patients and their relatives.

One advantage of the evaluation is the mixed-method design, which includes patient-reported outcomes, claims data from health insurance funds, and qualitative analyses of the SCNs’ project diaries. To evaluate the effects of continuous SCN support in Module 1, a prospective, non-randomized, multicenter, longitudinal study design was applied. Our patients were not assigned randomly, due to the limitation of the SCNs, who were trained and supported by the BKK insurance fund. Since randomization was not carried out, we are concerned about selection bias. Therefore, the baseline characteristics will be compared and we will control for baseline imbalance by using propensity score in our analysis. The propensity score with the inverse probability of treatment weighting (IPTW) method will be applied because the results from this method are similar to a randomized trial [23, 24]. For Module 2, we will match the intervention and control cases in order to reduce bias and confounding factors as much as possible. Moreover, given the severity of the patients’ conditions, we aimed to reduce the interview time by using the short versions of the instruments, if available, rather than applying generic instruments. In conclusion, we believe our study provides healthcare support for cancer patients. Hopefully, it can be applied for other severe chronic diseases. The evaluation of OSCAR is currently ongoing. In terms of providing healthcare support according to the patient’s needs, we expect our study to show that SCN support has a positive effect on the patient’s quality of life, health literacy, and participation in therapy planning.

## Supplementary information

**Additional file 1.** ICD- and OPS-Codes. ICD-10-Codes (summary of ICD-Codes with 5 digits). OPS-Codes.

## Data Availability

Not applicable

## Abbreviations

API-Dm: German modified version of the Autonomy Preference Index
DCS-10: Decision Conflict Scale
DRKS: German Clinical Trials Register
EORTC: European Organisation for Research and Treatment of Cancer
GEE: Generalized estimating equation
HLS-EU-Q6: European Health Literacy Survey
IPQ: Illness Perception Questionnaire
IPTW: Inverse probability of treatment weighting
MAR: Missing at random
MICE: Multiple imputation by chained equations
OSCAR: Oncological Social Care Project
OSSS-3: Oslo Social Support Scale
PRA-D: Patient Reaction Assessment
PRO: Patient-reported outcome
SCN: Social Care Nurse
SD: Standard deviation
SN: Study Nurse
