# Supplementary material for: Improving quality of life in cancer patients through higher participation and health literacy: study protocol for evaluating the oncological social care project (OSCAR)
Source: BMC Health Serv Res. 2019 Oct 26;19:754. doi: 10.1186/s12913-019-4585-0 (PMC6815389; doi:10.1186/s12913-019-4585-0)
Supplement: Supplementary file 1 — Additional file 1. ICD- and OPS-Codes. ICD-10-Codes (summary of ICD-Codes with 5 digits). OPS-Codes. [file 12913_2019_4585_MOESM1_ESM.docx]

**Additional file 1: ICD- and OPS-Codes**

**ICD-10-Codes (summary of ICD-Codes with 5 digits)**

- Leukemia (C91 – C92)
- Lymphoma (C82 – C86)
- Metastasized colorectal cancer (C18 – C20 (+C77 – C79))
- Malignant neoplasm of pancreas (C25)
- Malignant neoplasm of bronchus and lung (C34)
- Multiple myeloma and malignant plasma cell neoplasms (C90)
- Metastasized malignant neoplasm of breast (C50 (+C77 – C79))
- Metastasized malignant neoplasm of ovary (C56 (+C77 – C79))
- Metastasized malignant neoplasm of cervix uteri (C53 (+C77 – C79))
- Metastasized malignant neoplasm of corpus uteri (C54 (+C77 – C79))
- Malignant neoplasm of stomach (C16)
- Malignant neoplasm of esophagus (C15)
- Metastasized malignant neoplasms of lip, oral cavity, and pharynx (C00 – C14 (+C77 – C79))
- Metastasized malignant neoplasm of prostate (C61 (+C77 – C79))
- Metastasized malignant neoplasm of thyroid gland (C73 (+C77 – C79))
- Metastasized melanoma and other malignant neoplasms of skin (C43 – C44 (+C77 – C79))

**OPS-Codes**

- Surgical operation on the digestive tract (5-42 – 5-54)
- Surgical operation on the lymphatic tissues (5-402 – 5-404; 5-406)
- Radiotherapy, nuclear medicine therapy and pain management (8-52, 8-53, 8-91)
- Multimodal pain treatment, cytotoxic chemotherapy, complex treatment (8-541 – 8-544; 8-546; 8-918; 8-982; 8-98e)
